# Chronic Dietary Intake of Enniatin B in Broiler Chickens Has Low Impact on Intestinal Morphometry and Hepatic Histology, and Shows Limited Transfer to Liver Tissue

**DOI:** 10.3390/toxins10010045

**Published:** 2018-01-18

**Authors:** Sophie Fraeyman, Siska Croubels, Mathias Devreese, Richard Ducatelle, Michael Rychlik, Gunther Antonissen

**Affiliations:** 1 Department of Pharmacology, Toxicology and Biochemistry, Faculty of Veterinary Medicine, Ghent University, Salisburylaan 133, 9820 Merelbeke, Belgium; sophie.fraeyman@ugent.be (S.F.), mathias.devreese@ugent.be (M.D.); gunther.antonissen@ugent.be (G.A.); 2 Department of Pathology, Bacteriology and Avian Diseases, Faculty of Veterinary Medicine, Ghent University, Salisburylaan 133, 9820 Merelbeke, Belgium; richard.ducatelle@ugent.be; 3 Chair of Analytical Food Chemistry, Technical University of Munich, Alte Akademie 10, 85354 Freising, Germany; michael.rychlik@tum.de

**Keywords:** Enniatin B, broiler chicken, chronic dietary intake, carry-over, liver residue, intestinal toxicity, Chronic dietary intake of the *Fusarium* mycotoxin enniatin B in broiler chickens has a low impact on the intestinal morphometry. Above, histopathological examination of the liver did not reveal abnormalities and carry-over of enniatin B from feed into the liver was limited.

## Abstract

The *Fusarium* mycotoxin enniatin B (ENN B) is a so-called emerging mycotoxin frequently contaminating poultry feed. To investigate the impact of chronic ENN B exposure on animal health, broiler chickens were fed either a diet naturally contaminated with ENN B (2352 µg/kg) or a control diet (135 µg/kg) for 2, 7, 14, or 21 days. ENN B concentrations were determined in plasma and liver using a validated ultra-high performance liquid chromatography—tandem mass spectrometry UHPLC-MS/MS method. Liver was evaluated histologically, and the villus length and crypt depth of the duodenum, jejunum, and ileum were measured. Histopathology of the livers did not reveal major abnormalities. Feeding an ENN B-contaminated diet could possibly inhibit the proliferation of enterocytes in the duodenal crypts, but did not affect villus length, crypt depth, or villus length-crypt depth ratio of the jejunum and ileum. ENN B levels in plasma and liver were significantly higher in the ENN B-fed group and ranged between <25–264 pg/mL and <0.05–0.85 ng/g, respectively. ENN B carry-over rates from feed to liver tissue were 0.005–0.014% and 0.034–0.109% in the ENN B and control group, respectively. Carry-over rates were low and indicated a limited contribution of poultry tissue-derived products to the total dietary ENN B intake for humans. The above results support the opinion of the European Food Safety Authority stating that adverse health effects from ENN B in broiler chickens are unlikely.

## 1. Introduction

Enniatins (ENNs) are mycotoxins produced by a variety of *Fusarium* fungi, contaminating mainly grain and grain-based products. As reviewed by Fraeyman et al. (2017), enniatin A (ENN A), enniatin A1 (ENN A1), enniatin B (ENN B), and enniatin B1 (ENN B1) are the ENNs most frequently detected in feed, with a maximum prevalence ranging between 87% and 95%. Maximum contamination levels reported in feed were 1745, 2216, 1514, and 1846 µg/kg for ENN A, ENN A1, ENN B and ENN B1, respectively [1]. In a recent Danish study, ENN B was the most prevalent ENN, with all of the analyzed cereal samples (*n* = 110) being contaminated, and this was found in the highest concentration with a maximum contamination level of 3900 µg ENN B/kg in rye. In this study it was not specified whether the cereals were intended for human or animal consumption [2].

In vitro studies showed that ENN B is cytotoxic in different cell types, such as human adenocarcinoma colon (Caco-2) and human colon carcinoma (HT-29) cells, presumably due to its ionophoric properties. Moreover, in vitro studies suggest effects on endocrinology and immunity. In human adrenocortical carcinoma cells (H295R) ENN B reduced progesterone, testosterone, and cortisol secretion, and modulated the expression of genes involved in steroidogenesis. ENN B increased IL-10 secretion, interfered with dendritic cell migration, and decreased endocytosis by macrophages in vitro [1,3,4,5,6,7]. However, in vivo studies on the effects of dietary ENN B in livestock and companion animal are limited to poultry [1,7,8]. Feeding a multi-mycotoxin-contaminated diet including ENN A (28 µg/kg), ENN A1 (491 or 440 µg/kg), ENN B (12,716 or 11,233 µg/kg), ENN B1 (4057 or 3599 µg/kg), and beauvericin (BEA; 10,313 or 8926 µg/kg), for 14 days to broiler chickens or laying hens did not influence growth, feed intake and egg production [9].

Since the toxicokinetic properties of a mycotoxin determine the systemic exposure, such toxicokinetic studies are essential to evaluate the risks of dietary ENNs for human and animal health. Fraeyman et al. (2016) demonstrated that ENN B is poorly absorbed in broiler chickens after oral administration, with an absolute oral bioavailability of merely 11%. Furthermore, the total body clearance was high, namely 7.10 L/h/kg and 7.18 L/h/kg after intravenous injection (iv) and oral administration (po), respectively [10]. Although a low oral bioavailability was observed, it was also demonstrated that ENN B was readily distributed, since a volume of distribution of 33.9 and 29.4 L/kg was noted after iv and po administration, respectively. This can possibly lead to residues in edible tissues, such as liver. Due to the low oral bioavailability and the rapid clearance, systemic adverse health effects of dietary ENN B are unlikely in poultry [10]. This was also acknowledged by the European Food Safety Authority (EFSA), who identified no-observed-adverse-effect levels (NOAELs) of 763 and 674 µg ENN B/kg body weight per day for broiler chickens and laying hens, respectively [3].

On the other hand, both relatively high dietary exposure and the low oral absorption may lead to relatively high intestinal ENN B concentrations in broiler chickens, possibly leading to toxicity and impaired intestinal health. Previously, an influence on the intestinal mucus layer and epithelial antioxidative response in broiler chickens has been demonstrated for other *Fusarium* mycotoxins such as deoxynivalenol and fumonisins [11]. However, to the authors’ knowledge, no in vivo intestinal toxicity studies have been described before for the emerging ENN B. In vitro, ENN B induced cytotoxic effects in different intestinal cell lines. Low levels of ENN B (1.5–3 µM or 960–1920 µg/L) resulted in the generation of reactive oxygens species, lipid peroxidation, cell cycle arrest, apoptosis, and necrosis in Caco-2 cells, and reduction of transepithelial electrical resistance in intestinal porcine epithelial cells of the jejunum (IPEC-J2) [7,12].

Therefore, the aim of present study was to investigate the impact of feeding an ENN B-contaminated diet on the intestines and liver of broiler chickens, using morphometric and histological examinations, respectively. Moreover, an ultra-high performance liquid chromatography—tandem mass spectrometry (UHPLC-MS/MS) method was developed and validated to quantify ENN B in liver tissue, in order to calculate the transfer of ENN B from feed to liver tissue.

## 2. Results

### 2.1. Method Validation for Liver Tissue Analysis

The UHPLC-MS/MS method for the quantification of ENN B in liver was highly sensitive, with a limit of quantitation (LOQ) and detection (LOD) of 0.05 ng/g and 0.001 ng/g, respectively. A good linearity was observed, since the correlation coefficient was >0.9986 and the goodness-of-fit coefficient was 4.45%. There was no carry-over, since no peak higher than the LOD was detected at the elution zone of ENN B or the internal standard (IS) after injection of the reconstitution solvent. The results of the evaluation of the accuracy and precision fell within the acceptance criteria, and are summarized in the Supplementary Materials (Table S1).

### 2.2. Feeding Trial

**Figure 1:**
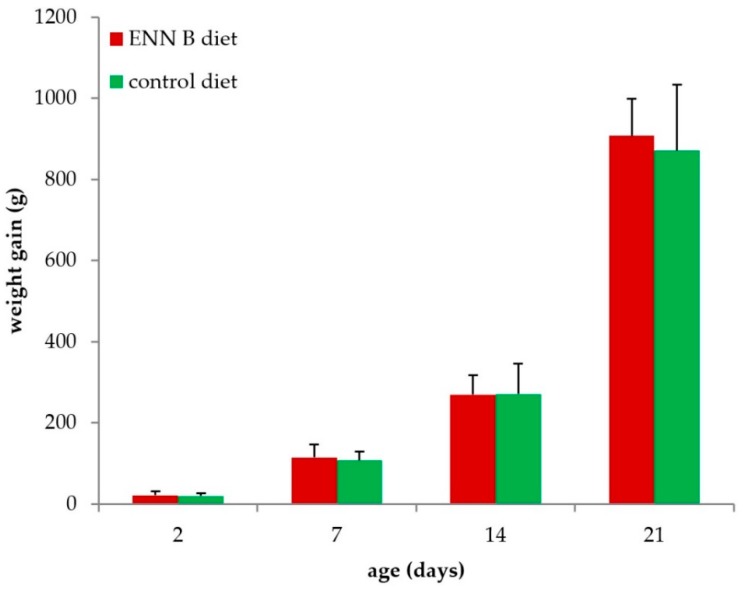
Body weight gain of broiler chickens fed the Enniatin B (ENN B)-contaminated diet and the control diet for 2, 7, 14, and 21 days. No significant differences in weight gain were observed between both diets (*n* = 8 in each group).

Feeding the ENN B-contaminated diet did not significantly affect body weight gain (*p* = 0.556, Figure 1), measured as the difference in body weight at the start of the experiment and at the age of euthanasia, over the age range studied. The average daily ENN B exposure in the ENN B-fed group was 396, 679, 312, and 316 µg ENN B/kg body weight/day at the age of 2, 7, 14, and 21 days, respectively.

Histopathological examination of the liver did not reveal abnormalities nor major differences between the ENN B-fed group and the control group (Supplementary Figure S1). Regarding intestinal toxicity, feeding the ENN B-contaminated diet did not significantly affect villus length, crypt depth or villus length-crypt depth ratio, except for crypt depth of the duodenum, which was smaller in the ENN B fed group (Table 1, *p* = 0.016). However, only a limited number of duodenal slides were available, which could bias the statistics, and therefore the statistically significant difference was probably not biologically relevant.

**Figure 2:**
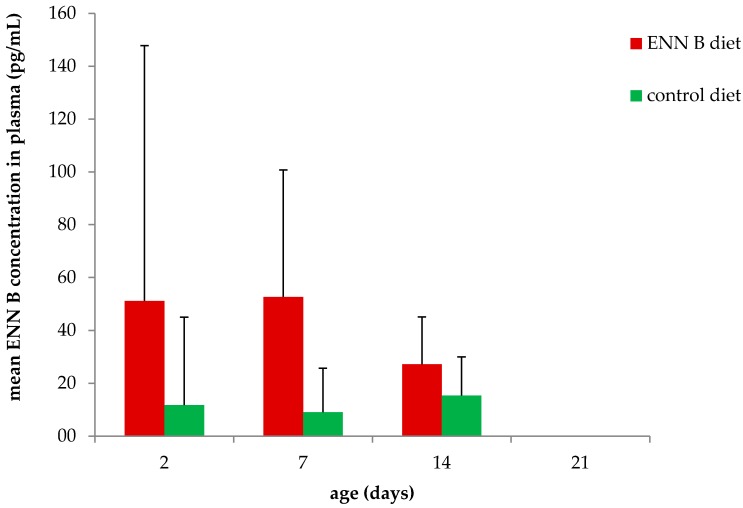
Mean (+SD) ENN B concentration in plasma of broiler chickens fed the ENN B contaminated diet and the control diet for 2, 7, 14, and 21 days (*n* ranged between 6–8 animals per group).

**Figure 3:**
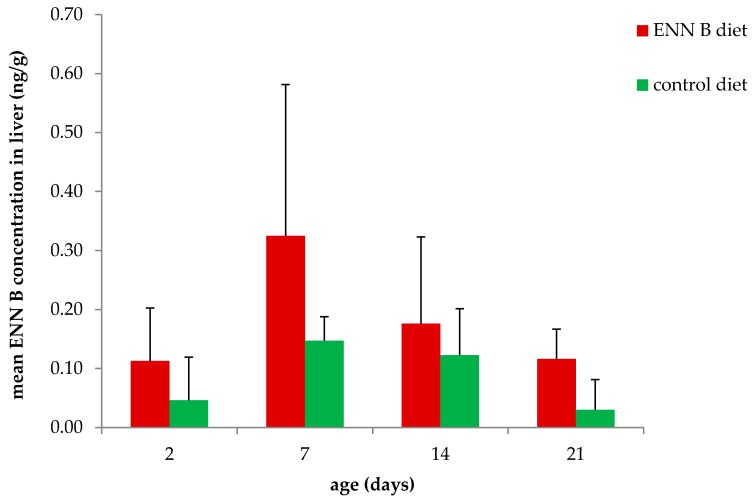
Mean (+SD) ENN B concentration in livers of broiler chickens fed the ENN B contaminated diet and the control diet for 2, 7, 14 and 21 days (*n* ranged between 7–8 animals per group).

ENN B concentrations in plasma (Figure 2) and liver (Figure 3) were significantly higher in the ENN B-fed group compared to the control in both plasma (*p* = 0.023) and liver (*p* = 0.003). ENN B levels ranged between <25–94 pg/mL and <25–264 pg/mL in plasma of broiler chickens fed the control and the ENN B-contaminated diet, respectively. ENN B levels in liver ranged between <0.05–0.29 and 0.05–0.85 ng/g in the control and ENN B group, respectively. Analysis of variance (ANOVA) showed no interaction between feed and age, thus the effect of the feed on the plasma concentration, liver concentration, villus length, crypt depth, and villus length-crypt depth ratio was independent of the age.

The ENN B carry-over from feed into liver ranged between 0.005–0.014% in the ENN B fed group and between 0.034–0.109% in the control group. Regarding carry-over, ANOVA showed an interaction between feed and age. Although still low, carry-over rate was significantly higher in the control group at the age of 7 and 14 days compared to the ENN B group (*p* < 0.01). Mean carry-over rates ± standard deviation were 0.014 ± 0.011 and 0.109 ± 0.030 at the age of 7 days for ENN B and the control group, respectively. At 14 days of age, mean carry-over rates ± standard deviation were 0.007 ± 0.006 and 0.091 ± 0.058% for the ENN B and control group, respectively.

## 3. Discussion

To the best of our knowledge, the effect of chronic dietary ENN B intake on intestinal and hepatic health of broiler chickens has not been thoroughly investigated before. Microscopic examination of the liver did not reveal major histopathological abnormalities. This is in accordance with the EFSA opinion since the highest mean daily ENN B exposure of 679 µg ENN B/kg body weight/day at the age of 7 days was still below the NOAEL of 763 µg ENN B/kg body weight/day [3]. The average daily ENN B exposure in the ENN B fed group was remarkably higher at the age of 7 days (679 µg ENN B/kg body weight/day), compared to the other age groups studied (312–396 µg ENN B/kg body weight/day). This explains the tendency of a higher ENN B liver concentration at 7 days of age, and this is related to the higher feed intake/kg body weight in younger animals, in conformity to the Ross 308 Broiler Performance Objectives [13]. To our knowledge, no other in vivo studies investigating hepatotoxic effects of ENN B have been published. A multi-parametric liver toxicity test using the human hepatocellular carcinoma cell line Hep-G2 demonstrated an acute hepatotoxic response from 0.9 µM ENN B (576 µg/L) onwards [2]. On the other hand, an IC_50_ value could not be calculated in Hep-G2 cells using the methylthiazoltetrazolium salt (MTT) assay at concentrations up to 30 µM (19,194 µg/L) [6]. These concentrations are a factor of 700 and 22,600 higher compared to the highest concentration found in the liver of the broiler chickens in the present study, respectively.

Accordingly, dietary ENN B did not significantly alter villus length, crypt depth or villus length-crypt depth ratio of the jejunum and ileum. Statistics showed a smaller crypt depth in the duodenum of the ENN B fed group, suggesting an inhibitory effect of ENN B on the proliferation of duodenal enterocytes. However, the number of histological slides examined was limited, and the difference is potentially not biologically relevant.

Although the contamination level of the feed was relatively high (2353 µg/kg), and despite the high volume of distribution of ENN B in broiler chickens (Vd up to 34 L/kg) [10], the ENN B concentrations found in plasma (up to 264 pg/mL) and liver (up to 0.85 ng/g) were low. This is in accordance with the low absolute oral bioavailability (F = 11%), the high total body clearance (Cl = 7 L/h/kg), and the biotransformation of ENN B in broiler chickens [10]. It should be mentioned that no extrapolation to other ENNs or other animal species can be made. As an example, a high oral bioavailability (F = 91%) for ENN B1 in pigs has been reported [14].

Callebaut et al. fed a multi-mycotoxin-contaminated diet to broilers and laying hens in order to determine residue levels in meat, liver, skin, and eggs [9]. Dietary contamination levels were 1710 or 2228 µg deoxynivalenol/kg, 488 or 606 µg HT-2 toxin/kg, 367 or 343 µg T2 toxin/kg, 753 or 820 µg zearalenone/kg, 41 or 66 µg 3-acetyldeoxynivalenol/kg, 91 or 227 µg 15-acetyldeoxynivalenol/kg, 28 µg ENN A/kg, 491 or 440 µg ENN A1/kg, 12,716 or 11,233 µg ENN B/kg, 4057 or 3599 µg ENN B1/kg, and 10,313 or 8926 µg BEA/kg in feed for broiler chickens or laying hens, respectively. Feeding the multi-mycotoxin-contaminated diet did not affect growth, feed uptake, nor egg production. Carry-over rates in liver were 0.16%, 0.12%, and 1.57% for ENN B, ENN B1, and BEA, respectively. In the present study, mean carry-over rates from feed to liver ranged between 0.005–0.014% in the ENN B-fed group, and 0.034–0.109% in the control group. Although still low, the carry-over rates were significantly higher for the control diet compared to the ENN B diet at the age of 7 and 14 days. Mean carry-over rates were 0.014% (ENN B) and 0.109% (control) at the age of 7 days, and 0.007% (ENN B) and 0.091% (control) at the age of 14 days. The higher carry-over rate in the control group could possibly indicate a saturation of absorption. However, this is in contrast with the study of Callebaut et al. who found a higher carry-over rate (0.16%) after feeding a more heavily contaminated diet. Furthermore, Callebaut et al. detected traces of ENN A and ENN A1 in meat, while traces of α-zearalenol were detected in both skin and liver. However, no other toxins than ENN B, ENN B1 and BEA could be quantified in these tissues. Highest mean concentrations reported by Callebaut et al. were 20.5 µg/kg (ENN B), 3.8 µg/kg (ENN B1), and 162 µg/kg (BEA) in liver, and 50 µg/kg (ENN B), 15 µg/kg (ENN B1), and 120 µg/kg (BEA) in skin. Carry-over rates in skin were 0.39% (ENN B), 0.37% (ENN B1) and 1.16% (BEA). In contrast, carry-over rates in meat were lower and ranged between 0.04–0.01% (ENN B), 0.041–0.025% (ENN B1), and 0.026–0.03% (BEA) [9]. In a Finnish survey, ENN B was found in turkey meat samples (up to 2 µg/kg) and in one out of 24 broiler liver sample (<0.4 µg/kg) collected from slaughterhouses. Mycotoxin concentrations in feed were not determined in this study. Therefore, it was not possible to determine the carry-over rates [15]. ENN B concentrations were not determined in meat samples in the present study. The concentrations reported in the liver samples in the present study are in line with those reported in the Finnish survey [15]. Although the carry-over rates of ENN B in liver vary, they are relatively low [9]. Besides, Finnish eggs (56–79%) and egg yolks (99.7%) were frequently contaminated with BEA and/or ENNs, but contamination levels were usually low. Maximum concentrations were higher in egg yolks compared to whole eggs and were 1.3 µg/kg, 7.5 µg/kg, 3.8 µg/kg, 7.0 µg/kg and 1.3 µg/kg for ENN A, ENN A1, ENN B, ENN B1 and BEA, respectively [16]. As determined by Callebaut et al., carry-over rates in eggs were 0.10%, 0.05% and 0.44% for ENN B, ENN B1 and BEA, respectively. Furthermore, Callebaut et al. quantified ENN B in a number of Belgian pig liver samples. However, no concentration or prevalence was reported. Thus no conclusions can be drawn regarding carry-over into pig-derived tissues [9].

Taken together, our data complement the EFSA opinion and suggest a low intestinal and systemic toxicity of ENN B in broiler chickens. The data on carry-over and residues of ENNs are limited, but nevertheless suggest a low contribution of ENNs from poultry-derived products to the total dietary ENN intake for humans. Since no extrapolation to other animal species can be made, future studies should focus on the toxicity, toxicokinetics and residues of ENNs and BEA in livestock animals other than poultry.

## 4. Materials and Methods

### 4.1. Chemicals, Products and Reagents

Analytical standard of ENN B was purchased from Fermentek (Jerusalem, Israel). The internal standard (IS) [^15^N_3_]-ENN B was synthesized according to Hu and Rychlik (2012) [17]. ENN B and the IS were stored at ≤−15 °C. Glacial acetic acid, water, methanol, and acetonitrile were of LC-MS grade (Biosolve, Valkenswaard, The Netherlands). Aceton was purchased from VWR (Leuven, Belgium). Millex-GN nylon filters (0.20 µm) were purchased from Merck-Millipore (Merck-Millipore, Overijse, Belgium).

### 4.2. Stock and Working Solutions

Stock solutions of ENN B (100 µg/mL) and the IS (10 µg/mL) for analytical experiments were prepared in methanol and stored at −20 °C. Working solutions of ENN B (ranging from 0.5–50 ng/mL) were prepared by appropriate dilution of the stock solutions in acetonitrile. These working solutions were used for the preparation of matrix-matched calibration standards and quality control samples and were freshly prepared for each analytical batch.

### 4.3. Experimental Diets

The feed was a custom-made wheat/rye (43%/7.5%) based mash diet, with soybean meal as the main protein source. Feed samples (500 g) were taken at three different locations in each batch, pooled, and analyzed with a validated LC-MS/MS method to quantitate aflatoxin B1, B2, G1 and G2, altenuene, alternariol, alternariol methylether, deoxynivalenol, 3-acetyldeoxynivalenol, 15-acetyldeoxynivalenol, diacetoxyscirpenol, ENN B, fumonisins B1, B2 and B3, fusarenon-X, neosolaniol, nivalenol, ochratoxin A, roquefortine-C, sterigmatocystin, T-2 toxin, HT-2 toxin, and zearalenone [18]. The decision limit (CCα) of each mycotoxin is summarized in the Supplementary Table S2 and was defined as the concentration at “the *y* intercept plus 2.33 times the standard deviation of the within laboratory reproducibility” (α = 1%), in the case of mycotoxins for which no maximum permitted limit has been established or only a maximum guidance level in feed has been recommended [19]. In the case of aflatoxin B1, CCα was established by spiking blank feed around the European maximum permitted limit of 0.02 mg/kg feed [20]. The corresponding concentration at the permitted limit plus 1.64 times the standard deviation of the within laboratory reproducibility equals the decision limit (α = 5%) [18].

The experimental ENN B diet was naturally contaminated with 2352 µg/kg ENN B and 160 µg/kg nivalenol, while the control diet was naturally contaminated with 135 µg/kg ENN B, 87 µg/kg nivalenol and 223 µg/kg deoxynivalenol. All other mycotoxins were below the corresponding CCα.

### 4.4. Feeding Trial

Sixty-four 1-day-old broiler chickens (Ross 308) of mixed gender were obtained from a commercial hatchery (Vervaeke-Belavi, Tielt, Belgium) and were randomly divided into two equal experimental groups. Each experimental group was housed in a different pen of 1.5 m^2^ with wood shavings. The environmental temperature was adjusted to the changing needs of the animals according to their age and an 18/6-h light/darkness program was applied. The animals had ad libitum access to drinking water and feed. Chickens were fed the ENN B contaminated diet or the control diet from day 1 until day 21. All animals were weighed at day 1, 2, 7, 14, and 21 and the feed intake per group was recorded daily. Using the mean body weight, feed intake and feed contamination level, the mean ENN B exposure per kg body weight was calculated for each age category. Eight animals of each group were euthanized on day 2, 7, 14, and 21 by intravenous injection of sodium pentobarbital (Natrium Pentobarbital 20%, Kela Veterinaria, Sint-Niklaas, Belgium) 3 h after the beginning of the light/darkness cycle. The total weight gain was defined as the difference in body weight between the start and the end of the experiment for each age category. The liver of each animal was collected to determine the effects of the diet using histology examinations. Accordingly, a sample of the duodenum, jejunum, and ileum of each animal was collected for morphometry. The samples were fixed in neutral-buffered formalin immediately after euthanasia, and were embedded in paraffin and stained with hematoxylin and eosin, using standard procedures. Liver was histologically evaluated by an independent pathologist. Due to technical problems, the number of histological slides of the duodenum was limited and was five or three, four, zero or two, and two or one for the ENN B or control group at the age of 2, 7, 14, and 21 days, respectively. Villus length and crypt depth of the duodenum, jejunum, and ileum were measured using a light microscope with Leica LAS software (Leica Microsystems, Diegem, Belgium). To determine ENN B concentrations in biological samples, blood and liver tissue samples were collected immediately after euthanasia. Blood samples were collected in heparinized tubes and centrifuged (2851× *g*, 10 min, 4 °C) and an aliquot of 250 µL plasma was stored at ≤−15 °C. Carry-over rate (%) was defined as the ratio of the ENN B concentration in the liver and the concentration in feed, multiplied by 100.

The animal experiment was approved by the Ethical Committee of the Faculties of Veterinary Medicine and Bioscience Engineering of Ghent University (EC 2013/170) on the ninth of January 2014.

### 4.5. Quantification of ENN B in Plasma and Liver

ENN B plasma concentrations were measured using a validated UHPLC-MS/MS method as described by Fraeyman et al. (2016) [10]. The LOD and LOQ for ENN B in plasma were 0.091 pg/mL and 25 pg/mL, respectively. In short, sample preparation consisted of deproteinization of plasma (250 µL) with acetonitrile (750 µL), followed by centrifugation (8517× *g*, 10 min, 4 °C). The acetonitrile phase was evaporated under a gentle nitrogen stream and the dry residue was reconstituted in 250 µL of acetonitrile/water (80/20, *v*/*v*). For liver tissue, 25 µL of a 25 ng/mL IS working solution was added to 1.0 g homogenized liver. Liver samples were extracted with 2 × 5 mL of acetone using a rotary mixer (Trayster digital, IKA^®^, Staufen, Germany, 70 rpm, 10 min) and centrifuged (2851× *g*, 10 min). The aceton phases were collected and evaporated under a nitrogen stream and the dry residue was reconstituted in 250 µL of methanol/water (90/10, *v*/*v*). After a vortex-mixing step, the sample was filtered through a Millex-GN nylon filter and transferred to an autosampler vial. A 5 μL aliquot was injected onto the UHPLC-MS/MS instrument.

The UHPLC-MS/MS system consisted of an Acquity H-Class UPLC coupled to a Xevo TQ-S mass spectrometer (Waters, Zellik, Belgium). The column used was a 50 mm × 2.1 mm i.d., 1.7 µm, C18 Acquity UPLC BEH column, with a 5 mm × 2.1 mm i.d. VanGuard BEH C18 guard column of the same material (Waters, Zellik, Belgium). Mobile phase A consisted of 0.1% glacial acetic acid in water, whereas mobile phase B consisted of acetonitrile. The following gradient elution program was run: 0.0–0.5 min (50% B), 0.5–6.0 min (linear gradient to 85% B), 6.0–8.0 min (85% B), 8.0–8.1 min (linear gradient to 50% B), 8.1–10.0 min (50% B). The flow rate was set at 300 μL/min. The column oven and tray temperature were set at 45 °C and 7 °C, respectively. The LC column effluent was interfaced to a Xevo TQ-S mass spectrometer (Waters, Zellik, Belgium) equipped with an electrospray ionization (ESI) probe operating in the positive ionization mode. Acquisition was performed in the multiple reaction monitoring (MRM) mode. Product ions with the highest signal intensity were used as quantitation ion. The following transitions (*m*/*z*) were monitored for qualification and quantitation, respectively, for ENN B: 640.4 > 214.2 and 640.4 > 196.2, and for IS: 643.3 > 215.3 and 643.3 > 197.1. Cone voltage and collision energy was set at 20.0 eV and 80.0 V, respectively.

### 4.6. Method Validation for Liver Tissue

The developed method was in-house validated based on the protocol described by De Baere et al. (2011) [21], using spiked blank liver samples obtained from healthy, untreated broiler chickens. Linearity, accuracy, precision, limit of quantitation (LOQ), limit of detection (LOD) and carry-over were determined in compliance with the recommendations and guidelines defined by the European Community and with criteria described in the literature [21,22,23,24,25].

#### 4.6.1. Calibration Curves

Matrix-matched calibration curves (range 0.05–5 ng/g) were prepared in 1.0 g of blank broiler chicken liver. The correlation coefficients (r) and goodness-of-fit coefficients (gof) were calculated and limits were set at ≥0.99 and ≤20%, respectively [21,24,25], with gof:

(1) $$\mathrm{gof}\;=\sqrt{\frac{\sum^{}\left(\%\;\mathrm{difference}\right)²}{n-1}}\;\mathrm{with}\;\%\;\mathrm{difference}\;=\;\frac{x_{\mathrm{back}\;\mathrm{calculated}}\;-\;x_{\mathrm{nominal}}}{x_{\mathrm{nominal}}}\;\times \;100$$

#### 4.6.2. Accuracy and Precision

Within-run accuracy and precision (repeatability) were determined by analyzing six blank samples that were spiked at 0.05, 0.5 and 5 ng/g in the same run. The between-run accuracy and precision (reproducibility) were determined by analyzing two blank samples spiked at 0.05, 0.5, and 5 ng/g on three different days (*n* = 6). The acceptance criteria for accuracy were: −50% to +20% and −30% to +10% for concentration levels ≤1 ng/g, and between 1 and 10 ng/g, respectively. The precision was evaluated by the determination of the relative standard deviation (RSD), which had to be below the RSDmax value. For the within-day precision, RSDmax is fixed at 30% and 25% for concentrations ≤1 ng/g and between 1 and 10 ng/g, respectively [23]. For between-run precision, the RSD had to be below the RSDmax value calculated by the Horwitz equation [23]. These RSDmax values were 71.0%, 50.2%, and 35.5% for concentrations at 0.05 ng/g, 0.5 ng/g, and 5 ng/g, respectively.

#### 4.6.3. Limit of Quantitation and Limit of Detection

The LOQ was the lowest concentration of the analyte for which the method was validated with an accuracy and precision that fell within the recommended ranges. Samples with a concentration below the LOQ were considered negative. The LOQ was also established as the lowest point of the calibration curve and was determined by analyzing six samples spiked at a concentration level of 0.05 ng/g, on the same day. The LOD was defined as the lowest concentration that could be recognized by the detector with a signal-to-noise (S/N) ratio of ≥3. The LOD values were calculated using the mean S/N of the blank samples spiked at the LOQ level.

#### 4.6.4. Carry-Over

Absence of carry-over was verified by analyzing the reconstitution solvent injected after the highest calibration sample (5 ng/g). If a peak was observed in the elution zone of ENN B or the IS, it had to be below the LOD.

### 4.7. Statistical Analysis

A two factor ANOVA was performed on weight gain, feed intake, plasma concentration, liver concentration, carry-over from feed to liver, villus length, crypt depth, and villus length-crypt depth ratio of the duodenum, jejunum, and ileum samples, using SPSS Statistics 23. Fixed factors were feed (ENN B and control) and age (2, 7, 14, and 21 days). The Levene’s test showed equality of error variances for all parameters, except for villus length of the jejunum, villus length of the ileum and crypt depth of the jejunum. Equality of error variances could be assumed when the logarithm of these parameters was calculated. Two factor ANOVA showed no interaction between feed and age, except for carry-over from feed to liver. The level of significance was set at 0.05.

## Figures and Tables

**Table 1:** Villus length, crypt depth and villus length-crypt depth ratio of the duodenum, jejunum and ileum of broilers after feeding a control or an ENN B-contaminated diet for 2, 7, 14, or 21 days. The mean of five measurements per segment per animal was calculated. Results are presented as the mean of eight animals per group ± standard deviation.

**Duodenum**						
**Age (Days)**	**Villus Length (µm)**		**Crypt Depth (µm) ^3^**		**Villus Length/Crypt Depth**	
	**ENN B**	**Control**	**ENN B**	**Control**	**ENN B**	**Control**
2	761 ± 207.9	976 ± 169.5	83 ± 15.0	109 ± 34.7	9 ± 2.3	12 ± 0.9
7	1283 ± 169.6	1303 ± 87.6	105 ± 32.6	137 ± 21.8	13 ± 4.6	10 ± 0.8
14	- ^1^	1526 ± 193.4	- ^1^	158 ± 6.7	- ^1^	10 ± 1.6
21	2109 ± 491.8	1780 ^2^	133 ± 7.5	158 ^2^	14 ± 1.2	11 ^2^
**Jejunum**						
**Age (Days)**	**Villus Length (µm)**		**Crypt Depth (µm)**		**Villus Length/Crypt Depth**	
	**ENN B**	**Control**	**ENN B**	**Control**	**ENN B**	**Control**
2	376 ± 34.6	389 ± 65.2	83 ± 11.2	87 ± 10.7	5 ± 0.6	5 ± 0.6
7	518 ± 225.8	561 ± 114.7	92 ± 13.3	122 ± 21.7	6 ± 0.6	4 ± 1.9
14	951 ^2^	932 ± 217.0	132 ^2^	142 ± 62.6	7 ^2^	7 ± 0.8
21	868 ± 232.6	887 ± 171.8	128 ± 17.6	134 ± 32.7	7 ± 2.4	7 ± 1.6
**Ileum**						
**Age (Days)**	**Villus Length (µm)**		**Crypt Depth (µm)**		**Villus Length/Crypt Depth**	
	**ENN B**	**Control**	**ENN B**	**Control**	**ENN B**	**Control**
2	322 ± 40.2	301 ± 56.9	80 ± 7.5	71 ± 15.6	4 ± 0.4	4 ± 0.8
7	509 ± 177.2	488 ± 81.1	105 ± 35.8	116 ± 15.2	5 ± 2.5	4 ± 0.3
14	613 ± 213.9	466 ± 123.2	123 ± 28.4	116 ± 43.6	4 ± 0.6	4 ± 0.9
21	598 ± 180.9	685 ± 133.1	165 ± 24.3	150 ± 32.2	4 ± 1.1	5 ± 1.3

^1^ Due to technical problems during preparation of the histological slides, data are unavailable; ^2^ or only a single measurement was possible; ^3^ Over the age ranges studied, no significant differences were found between the control diet and the ENN B diet (*p* range = 0.054–0.984), except for crypt depth of the duodenum (*p* = 0.016).

## Supplementary Materials

The following are available online at www.mdpi.com/2072-6651/10/1/45/s1, Table S1: Results of the Within- and Between-run Precision and Accuracy Evaluation for the Analysis of ENN B in Broiler Chicken Liver; Table S2: Decision Limit (CCα) of the Mycotoxins analyzed in the Experimental Diets; Figure S1: Histology of the liver of broiler chickens (20×).
