# Supplementary material for: Chronic Dietary Intake of Enniatin B in Broiler Chickens Has Low Impact on Intestinal Morphometry and Hepatic Histology, and Shows Limited Transfer to Liver Tissue
Source: Toxins (Basel). 2018 Jan 18;10(1):45. doi: 10.3390/toxins10010045 (PMC5793132; doi:10.3390/toxins10010045)
Supplement: Supplementary file 1 [file toxins-10-00045-s001.pdf]

Supplementary Materials: Chronic Dietary Intake of Enniatin B in Broiler Chickens Has Low Impact on Intestinal Morphometry and Hepatic Histology, and Shows Limited Transfer to Liver Tissue

Sophie Fraeyman, Siska Croubels, Mathias Devreese, Richard Ducatelle , Michael Rychlik and Gunther Antonissen

Table S1. Results of the Within- and Between-run Precision and Accuracy Evaluation for the Analysis of ENN B in Broiler Chicken Liver.

Theoretical concentration (ng/g)	Mean concentration ± SD (ng/g)	Precision, RSD (%)	Accuracy (%)
0.05 [a]	0.04 ± 0.005	11.9	-13.3
0.05 [b]	0.04 ± 0.010	23.8	-13.3
0.50 [a]	0.49 ± 0.005	1.1	-3.0
0.50 [b]	0.52 ± 0.027	5.2	3.7
5.00 [a]	4.75 ± 0.054	1.1	-5.1
5.00 [b]	4.99 ± 0.243	4.9	-0.3

[a] Within-run; [b] between-run; SD: standard deviation; RSD: relative standard deviation. Acceptance criteria for accuracy were: -50% to +20% and -30% to +10% for concentration levels ≤ 1 ng/g and between 1 and 10 ng/g, respectively. The precision was evaluated by the determination of the relative standard deviation (RSD), which had to be below the RSD_{max} value. For the within-day precision, RSD_{max} is fixed at 30% and 25% for concentrations ≤ 1 ng/g and between 1 and 10 ng/g, respectively [23]. For between-run precision, the RSD had to be below the RSD_{max} value calculated by the Horwitz equation [23]. These criteria were 71.0%, 50.2% and 35.5% for concentrations at 0.05 ng/g, 0.5 ng/g and 5 ng/g, respectively.

Table S2. Decision Limit (CCα) of the Mycotoxins analyzed in the Experimental Diets [18].

Mycotoxin	CCα (µg/kg)
aflatoxin B1	6.75 [a]-11.75 [b]-21.75 [c]
aflatoxin B2	1.53
aflatoxin G1	1.93
aflatoxin G2	2.39
altenuene	4.54
alternariol	11.98
alternariol methylether	17.75
deoxynivalenol	60.61
3-acetyl-deoxynivalenol	4.90
15-acetyl-deoxynivalenol	3.07
diacetoxyscirpenol	0.67
enniatin B [d]	80.0
fumonisin B1	31.84
fumonisin B2	24.37
fumonisin B3	23.18
fusarenon-X	16.58
neosolaniol	8.60
nivalenol	36.22
ochratoxin A	3.44

rocquefortin-C	1.08
sterigmatocystin	4.75
T2 toxin	9.38
HT-2 toxin	9.23
zearalenone	17.85

[a] for a maximum level in feed of 5 µg aflatoxin B1/kg, [b] for a maximum level in feed of 10 µg aflatoxin B1/kg, [c] for a maximum level in feed of 20 µg aflatoxin B1/kg, [d] cut-off of 80.0 µg/kg for ENN B

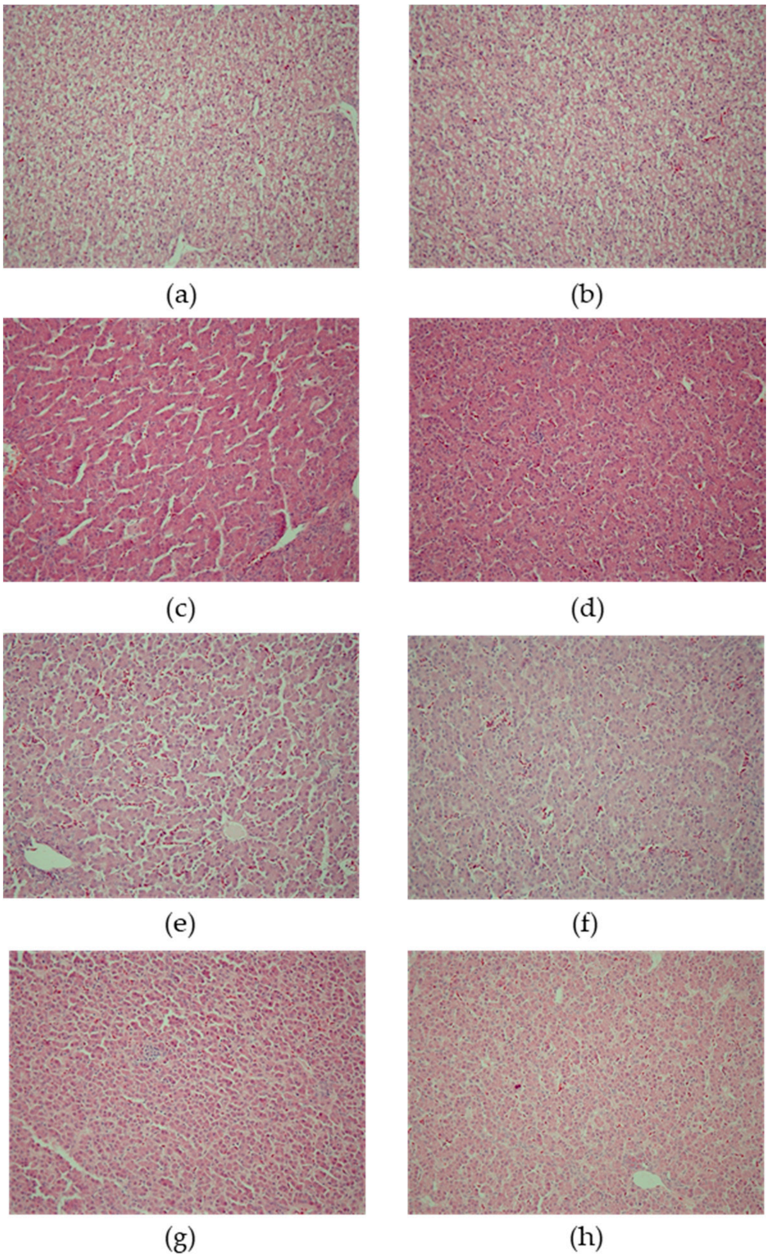

Figure S1. Histology of the liver of broiler chickens (20×). (**a**) ENN B diet, 2 days old; (**b**) control diet, 2 days old; (**c**) ENN B diet, 7 days old; (**d**) control diet, 7 days old; (**e**) ENN B diet, 14 days old; (**f**) control diet, 14 days old; (**g**) ENN B diet, 21 days old; (**h**) control diet, 21 days old.
